# Mining Facebook Data of People with Rare Diseases: A Content-Based and Temporal Analysis

**DOI:** 10.3390/ijerph15091877

**Published:** 2018-08-30

**Authors:** Laia Subirats, Natalia Reguera, Antonio Miguel Bañón, Beni Gómez-Zúñiga, Julià Minguillón, Manuel Armayones

**Affiliations:** 1 Eurecat, Centre Tecnològic de Catalunya, Unitat de eHealth, C/Bilbao, 72, 08005 Barcelona, Spain; 2 eHealth Center, Universitat Oberta de Catalunya, Rambla del Poblenou, 156, 08018 Barcelona, Spain; nataliareguera@gmail.com (N.R.); bgomezz@uoc.edu (B.G.-Z.); jminguillona@uoc.edu (J.M.); 3 Department of Philology, Almería University, Ctra. Sacramento, s/n, La Cañada, 04120 Almería, Spain; amhernan@ual.es

**Keywords:** social media, Facebook, rare diseases, data mining

## Abstract

This research characterized how Facebook deals with rare diseases. This characterization included a content-based and temporal analysis, and its purpose was to help users interested in rare diseases to maximize the engagement of their posts and to help rare diseases organizations to align their priorities with the interests expressed in social networks. This research used Netvizz to download Facebook data, word clouds in R for text mining, a log-likelihood measure in R to compare texts and TextBlob Python library for sentiment analysis. The Facebook analysis shows that posts with photos and positive comments have the highest engagement. We also observed that words related to diseases, attention, disability and services have a lot of presence in the decalogue of priorities (which serves for all associations to work on the same objectives and provides the lines of action to be followed by political decision makers) and little on Facebook, and words of gratitude are more present on Facebook than in the decalogue. Finally, the temporal analysis shows that there is a high variation between the polarity average and the hour of the day.

## 1. Introduction

In Europe, a disease is called rare (RD) when it has an incidence of less than five cases out of every 10,000 inhabitants. There are some 7000 known rare diseases, which according to the World Health Organization (WHO) estimates affect 7% of the world’s population [1]. That is, there are more than three million people affected by a RD in Spain alone. The magnitude of the problem is huge given that these pathologies are characterized by

- Early onset in life (two out of three appear before the age of two);
- Chronic pain (one in five patients);
- The development of motor, sensory or intellectual deficit in half of the cases, which give rise to a disability in autonomy (one in three cases); and
- In almost half of cases, a vital prognosis is at stake, since rare diseases have a rate of 35% of deaths before one year, 10% between one and five years and 12% between five and fifteen years.

There are several associations about rare diseases, such as the Spanish Federation of Rare Diseases (in Spanish, Federación Española de Enfermedades Raras or FEDER); the European Organization for rare diseases (EURODIS); the Ibero-American Alliance of Rare or Uncommon Diseases (in Spanish, Alianza Iberoamericana de Enfermedades poco frecuentes or ALIBER); the National Organization for Rare Diseases (NORD) in the US; and Raregenomics (http://www.raregenomics.org).

This research analyzed Facebook data in Spanish related to rare diseases. The analysis of these social networks had two objectives: (1) to help users interested in rare diseases, enabling them to maximize the engagement of their posts; and (2) to help organizations to align their priorities with the interests of people expressed in social networks.

In the first objective, an example of helping users to maximize the engagement of their posts is advising them to post their messages at a certain hour of the day. Facebook engagement is defined as the sum of comments, shares and reactions. Reactions are the six ways to react to posts with animated emotions: love, haha, wow, sad, angry, and the classic like. Another example is to advise them to adapt the content of their posts, including photos or a positive message, to maximize the number of likes or shares.

Regarding the second objective, FEDER was founded in 1999 with the aim of publicizing the RD, to serve as a link with the Administration and to fight for the normalization of the collective. To establish the lines of work, they have a decalogue of priorities [2], which serves for all local associations to work on the same objectives. Therefore, this document provides the lines of action to be followed by political decision-makers to defend, promote and improve the quality of life of patients with rare diseases in Spain. This decalogue has been elaborated in alignment with the concerns of the collective, but it must remain alive to ensure its effectiveness. To do so, it must be guaranteed that it reflects social reality. Recently, the rise of social networks has been generating a volume of information that can be very useful to understand the concerns of the RD collective. The objective of this work was to study whether the conversations in Facebook Groups are aligned with the priorities of the FEDER. The aim of this comparison was to establish whether there are any improvements that can be done in FEDER’s decalogue to reflect the social reality shown in the FEDER groups. Furthermore, this might help FEDER to understand the best way to generate engagement, which would help to extend the social dissemination of RD knowledge.

There are several works about social networks in RD. Davies [3] explained the insights of rare diseases from social media surveys, treating a specific rare disease and reviewing its advantages and limitations using social media surveys. The main advantages have been the use of on-line surveys, the ease of administration and low social desirability bias and participant engagement, while the main disadvantages have been the lack of objective participant assessment, the lack of opportunity for clarification, response bias and malicious responses.

Another interesting research study is [4], in which the authors states that, although health professionals use social media to gather information, they are skeptical of their value in communicating with patients. The current work is an extension of the preliminary analysis described in [5], which characterizes posts of the Spanish Federation of Rare Diseases (FEDER in Spanish). We found that the decalogue should focus more on help, life, people and children and less on disability, professionals and diseases. However, in that study, we did not perform a temporal analysis of the data. Reguera [6] performed a preliminary study in Spanish finding that there was statistical significance between the polarity of the message and the hour it was posted. However, no deeper conclusions were extracted.

Rareconnect (EURODIS), for example, is a platform where communities are created according to diseases or discussion groups of RD. Another platform is Crowdmed, a collaborative platform where patients explain their story (not their diagnosed disease or chronic symptoms) and a professional network gives information about its solution. #LoweResearchProject is a project which seeks to extract knowledge about the Lowe disease from information from social networks. Rooney [7] showed how to promote social networks to broadcast RD, while Chempetitive Group [8] showed how social networks are used to recruit patients to test new drugs. Finally, Smale [9] listed the most influential people from social networks about RD.

In addition, there are numerous studies that exploit the information of social networks in health. For example, Armayones, M; Requena, S; Gómez-Zúniga, B; Pousada, M; Banón, A.M. [10] confirmed that the use of social networks is not only extended by associations dedicated to RD, but also that its use is key to alleviate social isolation. In addition, Subirats et al. [11] and Subirats et al. [12] created new social platforms where doctors, patients and family members interested in neurological diseases can interact.

Regarding text mining in health science, it is used in several research studies. For instance, Palomino et al. [13] tried to understand the impact of social networks through feelings analysis in relation to attention deficit. They made recommendations to disseminate information better by changing the framing of the messages.

Larsen et al. [14], based on information collected from Twitter in real time, analyzed the mood of people with mental problems and understood their dispersion. They observed a correlation between the expression of emotions and the rates of suicide or burden of anxiety. Yang et al. [15] extracted information from forums with health topics, making an analysis of feelings. Their experiments show that conLDA outperforms the original Latent Dirichlet Allocation (LDA), and can cluster relevant medical terms and relevant questions together. Lasker et al. [16] analyzed the messages exchanged in a mailing list oriented to patients with a rare disease. The conclusions are that messages are rather biomedical than socioeconomic or organizational. In addition, it is found that the Internet provides a value to people with rare diseases to find people like them. Finally, Jacobs et al. [17] focused on monitoring the activity of several groups on Facebook related to congenital anomalies and concluded that, to improve the care received by families and patients, it is necessary for medical personnel to be involved in the social network.

Sentiment analysis can be used to evaluate public health systems. There are three main types of sentiment analysis methods [18]: knowledge-based, statistical and hybrid. The first one tries to classify text to identify emotions such as happiness, sadness or boredom, given that usually there are words that have a close relationship with emotions such as the aforementioned. On the other hand, statistical methods use machine learning techniques such as latent semantic analysis or support vector machines for improving emotion detection and recognition. Finally, hybrid approaches combine the use of machine learning techniques and ontologies to provide more context. In [19], sentiment analysis is used to evaluate the experience of users of the English public health system. Through a webpage, users have the possibility to evaluate the system, both quantitatively and with written response. It is concluded that the analysis of feelings can correctly predict the polarity of the messages, comparing it with the qualitative evaluation of the users. On the other hand, other studies (e.g., [20]) detect the intention and intensity of feelings on social networks. Experiments from five social networks demonstrate the effectiveness of the proposed Feeling Distinguisher system, which is a combination of supervised Latent Dirichlet Allocation (sLDA), Latent Dirichlet Allocation, and SentiWordNet. Finally, Akay et al. [21] proposed a framework to identify user communities and influential users for ascertaining user opinions of cancer treatment. They focused on positive and negative sentiments, together with the side effects of the treatment. Their objective was to identify communities to discover opinions of cancer treatment.

When analyzing data content, there are several measures used in the literature to compare the corpus of data: log-likelihood (LL) [22] is one of the most popular ones, together with DIFF [23], Bayes factor (BIC) [24], Effect size for Log Likelihood (ELL) [25], relative risk, log ratio or odds ratio. On the other hand, regarding summarization of content in word clouds, Diakopoulos et al. [26] provided a method for comparing two word clouds and gives as a recommendation to use 500 words to display on a standard computer screen (1150 × 550 pixels). For instance, Scanfeld et al. [27] used twitter data to analyze whether there is misuse of antibiotics in cases such as “flu” or “cold”. A word cloud (150 terms) is used to show the results.

Having analyzed that the existing literature does not show the alignment between the priorities of rare disease associations and social networks and that they do not give recommendations to users about how to maximize their engagement in terms of type of posts, content and time of the post, we performed this study to contribute to this field.

The rest of the paper is organized as follows. Section 2 describes how Facebook data were obtained and the methods for their evaluation. Section 3 presents the results obtained and their usefulness. Finally, conclusions and future work are drawn in Section 4 and Section 4.1, respectively.

## 2. Materials and Methods

Around 3900 Facebook posts (N=3917) were collected about rare diseases using Facebook Netvizz. As users’ information can be sensitive, guidelines provided by Townsend and Wallace [28] were followed for this social media research. The research groups analyzed are public (which means that they belong to Facebook pages openly available to the public), users’ anonymity and privacy are preserved in the study, and information is only displayed in an aggregated way, removing names and email addresses. The selection criterion was the following:

1. A pre-selection of groups was carried out from the Facebook search engine, containing the words in Spanish of “Syndrome” or “Association of”.
2. The first 10 groups of each of the two searches were preselected.
3. The groups had to comply with the following terms: they are Spanish associations, they have presence in FEDER (i.e., representing one or more rare diseases), they have more than 300 participations, they have at least 10 different people participating, they have groups in Spanish, and they affect children.

From the first search group (Syndrome), five groups were selected. However, from the second search group (Association of), no groups were selected because they did not fulfill the requisites explained above. The five groups selected were the Spanish Association for Research and Support of Worfam Syndrome (in Spanish “Asociación Española para la Investigación y Apoyo al Síndrome de Worfam”), Moebius syndrome Spain (in Spanish “Síndrome de Moebius España”), Spanish Association of Cyclic Vomiting Syndrome (in Spanish “Asociación Española del Síndrome del Vómito Cíclico”), Spanish Association of Marfan Syndrome (in Spanish “Asociación Española de Síndrome de Marfan”) and Syndrome 5P or CRI du Chat (in Spanish “Síndrome 5P o CRIT du Chat”). Then, Facebook Netvizz was used to download the data.

Two types of analysis were done with this data: content-based and temporal. In the content-based analysis, the following were applied using R and Python: (1) a description of the number of posts; (2) a generation of word clouds; (3) a comparison of texts; and (4) sentiment analysis. Regarding temporal analysis, the temporal evolution was performed using R.

Word clouds were generated using R. The darker and bigger are the words, the more frequently they appear. In Facebook data, we divided our data into three groups according to the engagement value of each post. Our goal was to see whether words with a high engagement are different from those with a low engagement. We considered “low engagement” to be the lower 33% of values of engagement; analogously, we considered "high engagement" to be the higher 33% of values and “medium engagement” to be the rest of the values between low and high engagement. Therefore, word clouds were generated for four datasets: one for those having a high, medium and low engagement, as well as an additional one for the decalogue. The engagement word clouds were created on groups of words which were selected based on the term frequency-inverse document frequency (Tf-idf) measure to disregard the frequent words that appear on all levels of engagement. Tf-idf is the most popular applied weighting scheme in some studies [29].

Facebook data were compared to the FEDER decalogue [2] to understand how different they are. The decalogue must remain alive and updated to ensure its effectiveness. For this reason, its comparison with Facebook data is relevant because it ensures that it reflects social reality. To make this comparison, LL was chosen to compare among different texts because common words are highlighted. The reason of this choice was because LL puts more emphasis in common words for a given topic than other measures [30], which are most likely to be used by non-expert users. LL was computed by constructing the contingency table of Table 1. Then, we computed LL as follows:

(1) $$E1=\frac{c(a+b)}{\left(c+d\right)}$$

(2) $$E2=\frac{d(a+b)}{\left(c+d\right)}$$

(3) $$LL=2(a\ln\frac{a}{E1}+b\ln\frac{b}{E2})$$

E1 and E2 are odds-ratio measures and they can be seen as the “strength” of a given word in one dataset while also considering its popularity in the other dataset. Then, LL measures whether a word is likely to appear in both Facebook or the decalogue, while also considering its frequency. LL is larger for words that appear in one dataset more frequently than they do in the other one.

Sentiment analysis of the content was performed using the Python library TextBlob. In sentiment analysis, both the polarity and the subjectivity were analyzed, the former being one of the most popular measures [31], although the latter is supposed to be more useful for analyzing emotions. To perform sentiment analysis, TextBlob Python library was used after translating text to English. This library is backed on Pattern (Natural Language Toolkit (NLTK)) [32] which is widely used by several universities. This toolkit uses statistical approaches and regular expressions to determine the polarity and subjectivity of a given text. The values are based on the adjectives of the text and they are optimized based on the frequency of the adjectives and successive words. The sentiment analysis library returns two attributes when analyzing a text: polarity and subjectivity. The polarity score is a real number within the range [−1.0, 1.0], where −1.0 is a negative text, 0 is a neutral text, and 1.0 is a positive text. On the other hand, subjectivity is a real number within the range [0.0, 1.0], where 0.0 is very objective and 1.0 is very subjective.

## 3. Results and Discussion

As explained in the Materials and Methods Section, results are divided into content-based and temporal analysis.

### 3.1. Content-Based Analysis

Firstly, the numeric and categorical variables of the Facebook dataset are described. In Table 2, there is a description of the numerical attributes of likes, reactions and engagement. It should be highlighted that the type of post with highest number of likes, reactions and engagement are photos; the status is type of post with the highest number of comments; and the events are the type of post with highest number of shares. These results are consistent with the general behavior in social networks that highlight the fact that Facebook posts in branded pages with photos are more prone to be liked [33,34].

**Figure 1:**
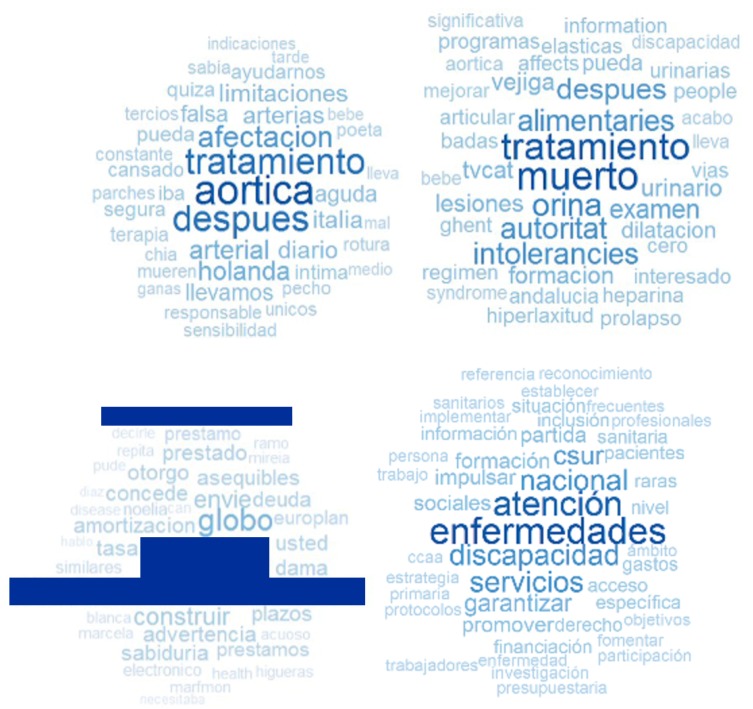
Word clouds of high (**top-left**), medium (**top-right**) and low engagement (**bottom-left**); and decalogue (**bottom-right**). Names and email addresses of the low engagement figure have been removed due to privacy issues.

To analyze the relation engagement–posts, we divided our data into three groups according to the engagement value of each post. Our goal was to see whether words with a high engagement are different from those with a low engagement. Therefore, word clouds were generated for four datasets: one each for those which have a high, medium and low engagement, and one for the decalogue. The engagement word clouds were created on group of words which were selected based on the term frequency-inverse document frequency (Tf-idf) measure to disregard the frequent words that appear for all levels of engagement. We could also see the word cloud of the decalogue in Figure 1. For high engagement, there are words such as “aortica”, “tratamiento”, “después”, “afectación” and “arterial” (in English, aortic, treatment, after, affectation and arterial, respectively). For medium engagement, there are words such as “tratamiento”, “muerto”, “orina”, “intolerancia” and “autoridad” (in English, treatment, dead, urine, intolerance and authority, respectively). For low engagement, there are words such as name, e-mail, “construir”, “globo” and “plazos” (in English, name, e-mail, build, balloon and terms, respectively). In the decalogue, there are words such as “enfermedades”, “atención”, “nacional”, “discapacidad”, “servicios” and “csur” (in English, diseases, attention, national, disability, services and csru (centers, services and reference units)). It is interesting to note that most of the words with low engagement belong to a spam message posted six times in intervals of 5 min. This is undoubtedly an unsolicited message with commercial intentions, trying to sell loans to people in need.

Then, we compared the Facebook words with the decalogue words using a log-likelihood (LL) measure. The higher the LL, the greater the relative difference between the apparitions between both texts. The top 12 words with highest LL are displayed in Table 3 and the words with the lowest LL score are displayed in Table 4.

To correctly interpret the results with a high LL, it is necessary to know in which of the corpuses has the word appeared most frequently. We can see, for example, that disability is more frequent in the decalogue, and yet help is more prevalent on Facebook.

The next step is to find correlations between polarity and subjectivity and the other attributes. To understand better the polarity and subjectivity in this domain, some examples of polarity and subjectivity scores are given in Table 5.

In Table 6, the attributes with an absolute Spearman correlation greater than or equal to 0.1 are highlighted in bold, which are polarity–likes, polarity–reactions, subjectivity–comments, and subjectivity–engagement. Correlations are significant (*p*-value < 0.05) except for the correlation between subjectivity and the number of shares.

### 3.2. Temporal Analysis

**Figure 2:**
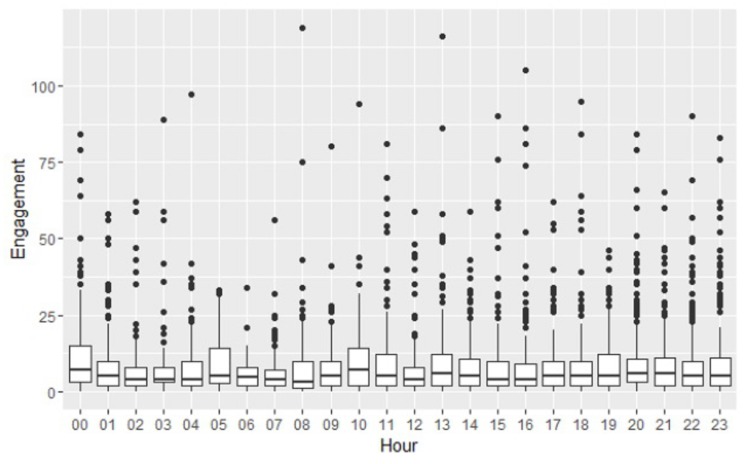
Engagement vs. hour.

To perform the temporal analysis of the Facebook data, we extracted the timestamp in which the post was issued, and used it to get the day of the week and the hour. With these data, we established whether there is any relationship between them and polarity or engagement. In Figure 2, we show the relationship between engagement and hours of the day. We can see that the times with the highest engagement are midday and before going to sleep.

**Figure 3:**
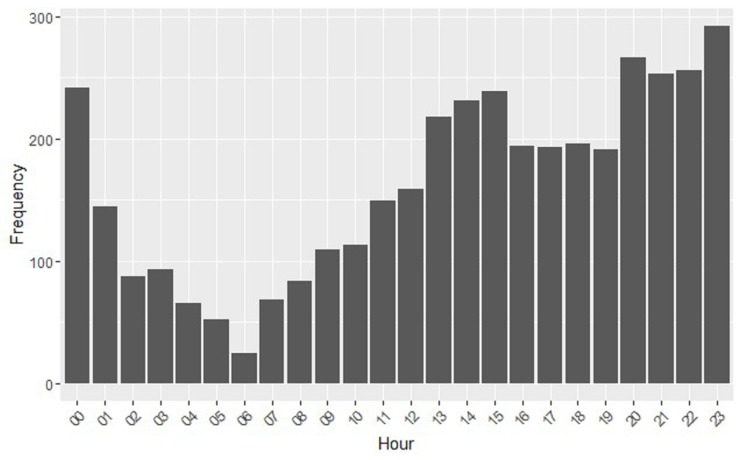
Frequency vs. hour.

On the other hand, in Figure 3, we show the frequency of appearance of the posts by hour of the day. We observe that there are two bands in which they are issued more frequently, during 13:00–15:00 and 20:00–00:00. These hours coincide with those of greater free time and justify a greater activity. This also highlights how, as the night progresses, the number of posts decreases, reaching its minimum at 6:00. When we performed an analysis of variance (ANOVA), we obtained the following values: polarity–day (0.158), polarity–hour (0.002), engagement–day (0.418) and engagement–hour (0.828).

**Figure 4:**
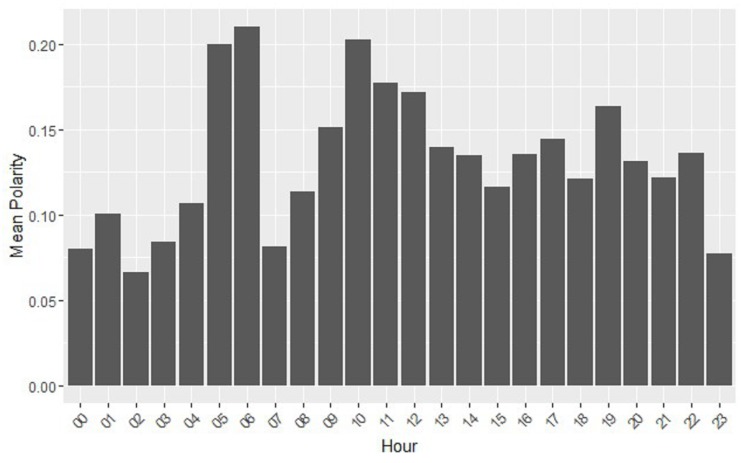
Mean polarity vs. hour.

In this study, we considered intervals of time such as the morning (6:00–12:00), afternoon (12:00–18:00), evening (18:00–24:00) and night (24:00–6:00). Using those time intervals to analyze polarity, we could see that the highest mean polarity is for the morning, which then progressively decreases during the afternoon, evening and night. With reference to the analysis of the polarity vs. the day, the top polarity goes for Wednesday, and then progressively decreases on Thursday, Friday, Tuesday, Monday, Saturday and Sunday. Figure 4 shows the mean polarity per hour; the hours with the highest polarity are the hours before going to work, 5:00–6:00, and the hours between 10:00 and 13:00.

**Figure 5:**
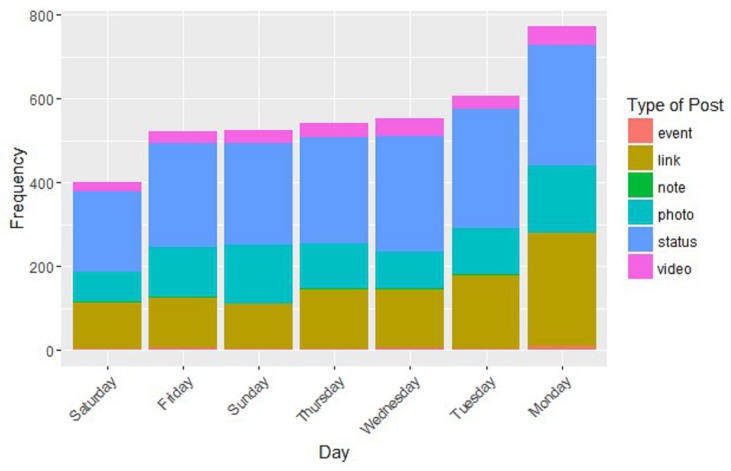
Day frequency vs. type of post.

Regarding engagement, the highest mean engagement is for the evening, which then decreases at night, morning and afternoon. Figure 5 shows the type of post also varies throughout the week. We note that the reason for the differences between Monday and the rest of days of the week is the number of link posts. In Figure 5, we also see that the days ordered from a higher to lower frequency of posts are Monday, Tuesday, Wednesday, Thursday, Sunday, Friday and Saturday. This pattern is consistent with other findings that show that users are most likely to connect when other users are connected, as communication among members is one of the main purposes of communities of social networks such as Facebook, especially for non-family relationships [35].

## 4. Conclusions

The main goals of this research have been to characterize both social media data such as Facebook engagement and the decalogue of the Spanish Federation of Rare Diseases (FEDER) datasets. The characterization is carried out considering a content-based and a temporal analysis. In addition, the Facebook dataset is compared with the decalogue of the FEDER and recommendations are given to users to maximize the engagement of their posts.

In the characterization of the Facebook data, we found similar results to the existing literature: photos are the type of posts with the highest number of reactions and engagement, while statuses are the type of posts with the highest number of comments, and events are the type of posts with the highest number of shares. Regarding the word clouds, we could see that the words that appear in Facebook are words of gratitude, while words that appear in the decalogue word cloud are related to diseases, attention, disability and services. We also found differences among word clouds grouped by level of engagement: the low engagement word cloud is related to spam messages, while medium/high engagement word clouds are related to treatments. As for correlations, we found that the attributes with the highest correlations (an absolute value above or equal 0.10) are polarity–likes, polarity–reactions, subjectivity–comments, and subjectivity–engagement.

With respect to the values obtained by the log-likelihood (LL), we observed that the following words have a large presence in the decalogue and are little-used on Facebook: disability, professionals and diseases. Similarly, the following are the words most present on Facebook with little representation in the decalogue: help, life, people and children. The analysis of the temporal variables has shown us that there is high correlation between the polarity and the time of day, which is not observed either with the engagement or with the day of the week.

Studying polarity and subjectivity in social networks has several practical applications. For example, an early detection of depression system can be built [36], or an early detection system of bipolar behavior. In [31], the authors stated that most sentiment analysis studies are focused on polarity, but subjectivity is also very important to detect “private states” (opinions, emotions, sentiments, beliefs, and speculations) [37].

Because of our study, the recommendations we give to users interested in rare diseases if they want to maximize their engagement of their posts are as follows: (1) use photos in their posts; (2) update their status; (3) post content of common interest; and (4) post at midday or late at night. Regarding the recommendations to FEDER, we recommend they focus more on concepts such as help, life, people and children.

### 4.1. Future Work

As for future work, there are several improvements that could be made. One is to improve the content-based analysis; a limitation of our current work is that only posts in Spanish were analyzed. In the future, other languages may be considered. In the same line, other associations of other countries different from FEDER may be considered. In addition, more Facebook groups will be analyzed. Concerning the improvement of the comparison of Facebook data with the FEDER decalogue, other initiatives such as the ones mentioned in Section 2 could be applied and compared. To improve the content-based analysis, sentiment analysis of the images could be performed. There are some application programming interfaces (APIs) to perform this, such as https://indico.io, which could allow us to obtain the polarity and subjectivity of images. From a language processing point of view, having such few data in the decalogue may cause the obtained results to not be reliable. For future work, a semantic analysis will be performed to compare results with LL measures. Finally, another interesting line of research would be to perform supervised and unsupervised learning to be able to predict engagement and determine profiles, respectively.

## Figures and Tables

**Table 1:** Contingency table used to calculate the log-likelihood (LL) ratio.

	Facebook	Decalogue	Total
Frequency of word	*a*	*b*	a+b
Frequency of other words	c-a	d-b	c+d-a-b
Total	*c*	*d*	c+d

**Table 2:** Characterization of the dataset mean (std).

Attribute	Event	Link	Note	Photo	Status	Video	Total
Likes	3.8 (4.3)	5.2 (6.1)	2.3 (3.2)	12.0 (12.8)	4.6 (6.5)	6.1 (6.4)	6.4 (8.6)
Comments	0.3 (0.6)	0.8 (1.9)	0.4 (1.0)	2.6 (5.3)	2.9 (5.9)	0.8 (1.8)	2.1 (4.9)
Reactions	3.9 (4.3)	5.3 (6.3)	2.3 (3.2)	12.2 (13.1)	4.7 (6.6)	6.1 (6.5)	6.5 (8.7)
Shares	4.1 (9.7)	0.0 (0.4)	0.0 (0.0)	0.9 (4.9)	0.1 (1.4)	0.4 (3.0)	0.3 (2.7)
Engagement	8.3 (9.5)	6.2 (6.9)	2.8 (3.6)	15.6 (17.4)	7.7 (10.3)	7.3 (9.4)	8.9 (11.8)
Instances	34	1063	9	792	1787	232	3917

**Table 3:** Top 12 words with the highest LL score.

Word	LL Score	Times in Facebook	Times in the Decalogue
nacional (national)	20.2	32	9
discapacidad (disability)	13.4	53	9
nivel (level)	7.8	28	5
ayuda (help)	7.5	195	1
profesionales (professionals)	6.1	23	4
referencia (reference)	5.0	28	4
vida (life)	4.3	190	2
frecuentes (frequents)	3.8	35	4
enfermedades (diseases)	3.3	192	12
ser (to be)	3.2	167	2
personas (people)	3.0	206	3
hijo (son)	3.0	112	1

**Table 4:** Words with the lowest LL score.

Word	LL Score	Times in Facebook	Times in the Decalogue
causa (cause)	0.063	48	2
social (social)	0.051	36	1
difundir (promulgate)	0.047	23	1
medio (middle/way)	0.031	24	1
experiencias (experiences)	0.027	34	1
forma (form)	0.021	52	2
general (general)	0.01	32	1
cuanto (how much)	0.01	26	1
todas (all)	0.007	91	3
dice (says)	0.000014	29	1

**Table 5:** Examples of polarity and subjectivity scores.

Text	Polarity	Subjectivity
Again, my daughter with her crises. This is already once a month, isn’t it dreadful to know that she can not be like the rest of her friends or brothers??	−1	1
Happy day Cri Du Chat dear family!!!	1	1
This article provides rehabilitation exercises for cerebellar ataxia think it may be interesting for patients with Wolfram.	0.5	0.5
I will attach separate interviews. The next one is aimed at parents.	0	0

**Table 6:** Correlations between polarity and subjectivity and the other attributes (N = 3917).

Attribute	Likes	Comments	Reactions	Shares	Engagement
Polarity	**0.10**	0.04	**0.10**	0.02	0.08
Subjectivity	0.07	**0.11**	0.07	0.03	**0.10**
